# Mechanism of Simiao Decoction in the treatment of atherosclerosis based on network pharmacology prediction and molecular docking

**DOI:** 10.1097/MD.0000000000035109

**Published:** 2023-09-08

**Authors:** Qian Li, Yihui Chai, Wen Li, Liancheng Guan, Yizi Fan, Yunzhi Chen

**Affiliations:** a Guizhou University of Traditional Chinese Medicine, Guiyang City, China; b The Second Affiliated Hospital of Guizhou University of Traditional Chinese Medicine, Guiyang City, China; c Chongqing High-tech Zone People’s Hospital, Chongqing City, China.

**Keywords:** atherosclerosis, molecular docking, network pharmacology, Simiao Decoction

## Abstract

To explore the molecular mechanism of Simiao Decoction (SMD) intervening atherosclerosis (AS). The main components and potential mechanisms of SMD remain unknown. This study aims to initially clarify the potential mechanism of SMD in the treatment of AS based on network pharmacology and molecular docking techniques. The principal components and corresponding protein targets of SMD were searched on Traditional Chinese Medicine Systems Pharmacology Database and Analysis Platform and the compound-target network was constructed by Cytoscape3.9.1. AS targets were searched on DrugBank, OMIM, and TTD databases. The intersection of compound target and disease target was obtained and the coincidence target was imported into STRING database to construct a protein–protein interaction network. We further performed Gene Ontology and Kyoto Encyclopedia of Genes and Genomes pathway enrichment analysis on the targets. The molecular docking method was used to verify the interaction between core components of SMD and targets. We created the active compounds-targets network and the active compounds-AS-targets network based on the network database containing Traditional Chinese Medicine Systems Pharmacology Database and Analysis Platform, DrugBank, OMIM, and TTD. We discovered that the therapy of AS with SMD involves 3 key substances—quercetin, kaempferol, and luteolin—as well as 5 crucial targets—ALB, AKT1, TNF, IL6, and TP53. The Gene Ontology and Kyoto Encyclopedia of Genes and Genomes enrichment analysis revealed that the shared targets involved a number of signaling pathways, including the advanced glycosylation end product-receptor of AGE signaling pathway in diabetic complications, Hepatitis B, Lipid and atherosclerosis, Chemical Carcinogenesis-Receptor Activation, and Pathways in Cancer. The molecular docking demonstrated that the binding energies of quercetin, kaempferol, and luteolin with 5 important targets were favorable. This study reveals the active ingredients and potential molecular mechanism of SMD in the treatment of AS, and provides a reference for subsequent basic research.

## 1. Introduction

Large and medium-sized arteries are the primary targets of the chronic illness atherosclerosis (AS).^[1]^ It is characterized by localized inflammation, lipid accumulation, smooth muscle cell proliferation, apoptosis, necrosis, and fibrosis. Moreover, AS provides the pathophysiological underpinning for certain cardiovascular illnesses. One of the most common diseases.^[2]^ The pathological process of AS mainly involves lipid deposition, endothelial damage, foam cell accumulation, and rupture of unstable plaques, which are prone to rupture and promote thrombosis, such as coronary artery disease and ischemic stroke.^[3]^ It’s the potential mechanism of cardiovascular adverse events.^[4]^ A number of pathophysiological processes in the body, including the inflammatory response, are linked to the development and progression of AS. Tumor necrosis factor α (TNF-α), proinflammatory factor interleukin (IL)-6, and AS can all be accelerated by inflammatory cytokines. Important clinical indications that represent the intensity of the inflammatory response in vivo are the levels of IL-1 and the anti-inflammatory cytokine IL-10.^[5]^ It is significant that traditional Chinese medicine has had outstanding outcomes in treating patients with various stages of AS while also significantly lowering the incidence of side effects and problems.^[6,7]^

The practice of traditional Chinese medicine, which continues to be important in preserving peoples’ health, is getting more and more recognition on a global scale. In order to treat complicated diseases like cardiovascular disease, cancer and diabetes, modern medicine has adopted the notion of traditional Chinese medicine and uses compound medications.^[8]^ What is noteworthy is that network pharmacology is a new field that analyzes biological networks using the systems biology theory, chooses certain signal nodes, and develops multi-target therapeutic molecules. The Simiao Decotion (SMD)-active ingredients-AS target network and SMD-AS common target protein–protein interaction (PPI) network were created using the network pharmacology method. Gene Ontology (GO) and Kyoto Encyclopedia of Genes and Genomes (KEGG) enrichment were used to analyze the biological process, cell components, molecular functions, and key pathways, among other things, to systematically elucidate the mechanism of SMD in the treatment of AS.

## 2. Materials and methods

### 2.1. Screening of active compounds and collection of targets

The compounds were searched by inputting “Huangqi,” “Danggui,” “Jinyinhua,” and “Gancao” as keywords on the Traditional Chinese Medicine Systems Pharmacology Database and Analysis Platform (TCMSP, https://tcmspw.com/tcmsp.php).^[9]^ Based on parameters of pharmacokinetics,^[10]^ oral bioavailability ≥ 30% and drug-likeness ≥ 0.18 were selected as the conditions for screening active compounds to determine the active compounds in SMD.^[11,12]^ All targets were introduced by the Uniprot database (https://www.uniprot.org/), and then a network of “Active compound targets” was built with Cytoscape 3.9.1 software.

### 2.2. Construction of active compound-AS-targets network

AS related targets were searched by entering “Atherosclerosis” as keyword in DrugBank (https://www.drugbank.ca),^[13]^ OMIM (https://omim.org/),^[10]^ and TTD (http://db.idrblab.net/ttd/). The Venn diagrams of the targets of SMD and AS were drawn out using R, and then the screening of common targets as important potential targets intervening AS in SMD was performed. The network of “active compound-AS-targets” was built using Cytoscape 3.9.1 software.

### 2.3. Construction of PPI network

In order to build a PPI network, we entered the targets intervening AS in SMD into the STRING.11.5 (https://string-db.org) platform with the mutual score set to “medium confidence 0.4” and the species set to “Human sapiens.”^[14]^ The topological parameters of the PPI network were acquired by the “Network Analysis” function in the Cytoscape 3.9.1 software, among which the greater degree value is the most important in the PPI network. The top 5 core targets were selected based on degree scores as important targets for SMD to interfere with AS. Then, the PPI networks of the 5 core targets were constructed by the STRING.11.5 platform and setting “Highest confidence 0.9” and the species “Homo sapiens.”

### 2.4. Biological function and pathway analysis

The GO function^[15]^ and the analysis of the KEGG pathway^[16]^ were performed for AS targets that intervene in SMD and “Homo sapiens” species in the DAVID 2021 database (https://david.ncifcrf.gov/home.jsp).^[17,18]^ The functions of the GO and KEGG pathways were subjected to visualization analysis using ggplot in the R package.^[19]^

### 2.5. Analysis of molecular docking between active compounds and the core protein receptor

The top 5 targets of the degree value in the PPI network were selected as protein receptors, and the active compounds of the top 3 degrees were selected as ligands of the active compounds in the SMD intervening in AS for verifying the molecular docking, respectively. The target protein files in PDB format and the active compound files in SDF were obtained from the RSCB PDB database (https://www.rcsb.org/)^[20]^ and the PubChem database.^[21,22]^ Chem Office software was used to perform mol2 format conversion and energy minimization in active compounds. After water molecules, original ligands, and polarized hydrogen from target proteins were removed via PyMOL software,^[23]^ the affinity and binding energy between the target protein and the active compounds were analyzed by Autodock Vina and Python scripts. Meanwhile, the binding energy (affinity) ≤ − 5.0 kJ/mol was a screening condition in this study.

**Figure 1.**
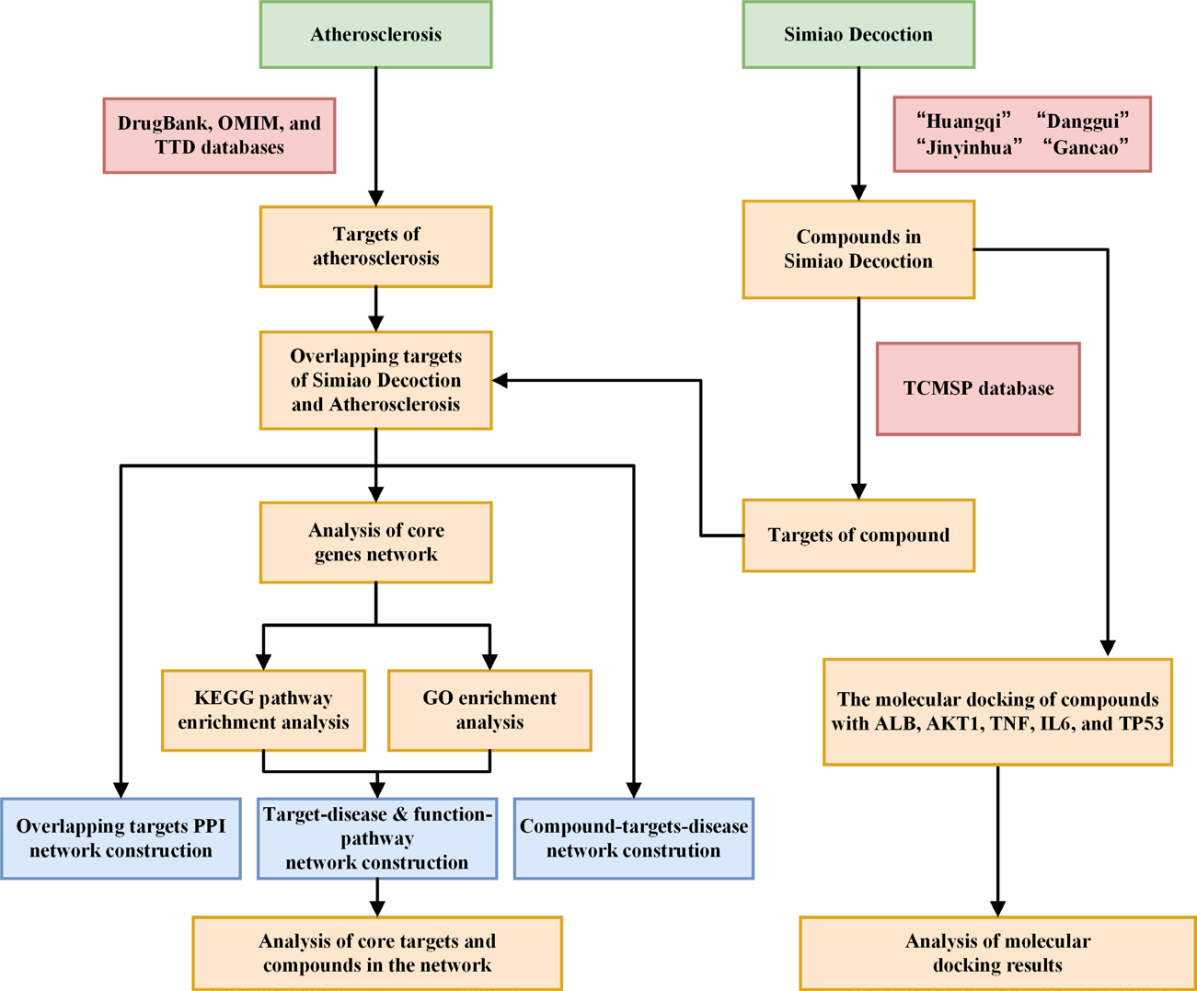
The workflow of mechanism of SMD in the treatment of AS. AS = atherosclerosis, SMD = Simiao Decotion.

The workflow of our study is shown by flowchart (Fig. 1).

## 3. Results

### 3.1. Active compounds and targets of SMD

Based on oral bioavailability ≥ 30% and drug-likeness ≥ 0.18, 137 active chemicals in SMD were chosen from the TCMSP database. A total of 124 active compounds were retrieved after comparison with the PubChem database, which resulted in the deletion of 13 active compounds (Table 1) due to the lack of matching compounds. The Uniprot database was used to determine the names of the target compounds in SMD, and the TCMSP was used to search for the matching 257 targets. After removing duplicates, there were still 257 targets.

**Table 1:** The list of 13 active compounds for which the corresponding target was not found.

MOL ID	Compound
MOL000374	5’-hydroxyiso-muronulatol-2’,5’-di-O-glucoside
MOL000398	isoflavanone
MOL000438	(3R)-3-(2-hydroxy-3,4-dimethoxyphenyl)chroman-7-ol
MOL002707	phytofluene
MOL003059	kryptoxanthin
MOL003062	4,5’-Retro-.beta.,.beta.-Carotene-3,3’-dione,4’,5’-didehydro-
MOL003101	7-epi-Vogeloside
MOL003108	Caeruloside C
MOL003124	XYLOSTOSIDINE
MOL004860	Licorice glycoside E
MOL004905	3,22-Dihydroxy-11-oxo-delta(12)-oleanene-27-alpha-methoxycarbonyl-29-oic acid
MOL004917	glycyroside
MOL005013	18α-hydroxyglycyrrhetic acid

### 3.2. Potential targets of SMD-intermediating AS

**Figure 2.**
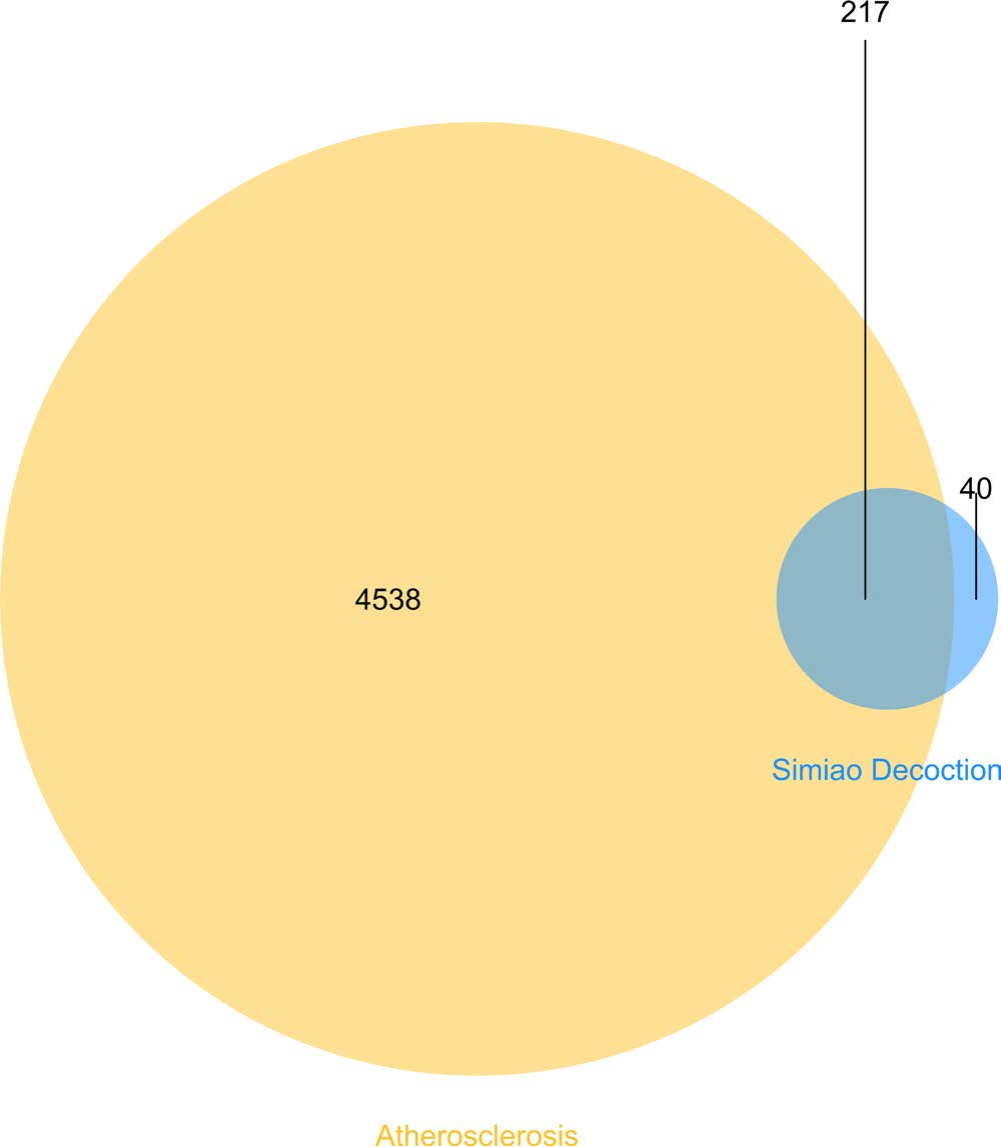
Venn diagram of SMD and the intersection genes in AS. AS = atherosclerosis, SMD = Simiao Decotion.

From DrugBank, OMIM, and TTD, respectively, searches were made for 4739, 3, and 35 known targets associated to AS. 4755 AS-related pathogenic targets were obtained after the duplicates were eliminated. The intersections of the active substance and the AS-related pathogenic 217 targets were discovered using the Venn diagram (Fig. 2).

### 3.3. Active compounds-AS-targets network.

**Figure 3.**
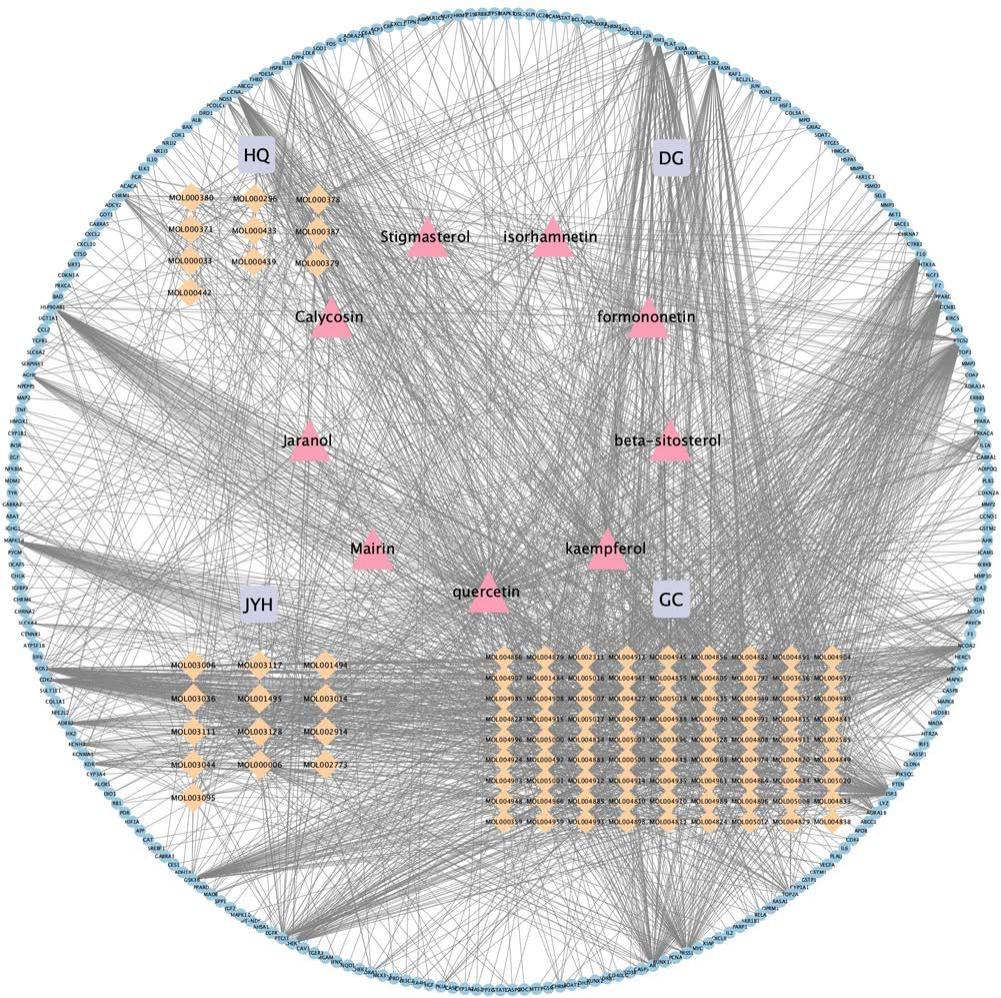
Active compounds – 257 targets network diagram. Notes: Blue represents targets, pink represents the common active compounds of the *Radix Astragali* (Huangqi-HQ), *Radix Angelicae sinensi*s (Danggui-DG), *Lonicerae Japonicae Flos* (Jinyinhua-JYH), and *Licorice* (Gancao-GC). Light orange represents other active compounds of *Radix Astragali* (Huangqi-HQ), *Radix Angelicae sinensis* (Danggui-DG), *Lonicerae Japonicae Flos* (Jinyinhua-JYH), and *Licorice* (Gancao-GC).

Related targets of intervening Cytoscape 3.9.1 software was used to construct and analyze the active compound-target connection in SMD, and the resulting “active compounds-257 targets” network diagram is depicted in Figure 3. There are 374 nodes (257 targets, 124 active compounds) and 2165 edges in the figure where circles in blue represents targets, pink represents the common active compounds of the *Radix Astragali* (Huangqi-HQ), *Radix Angelicae sinensi*s (Danggui-DG), *Lonicerae Japonicae Flos* (Jinyinhua-JYH), and *Licorice* (Gancao-GC).

**Figure 4.**
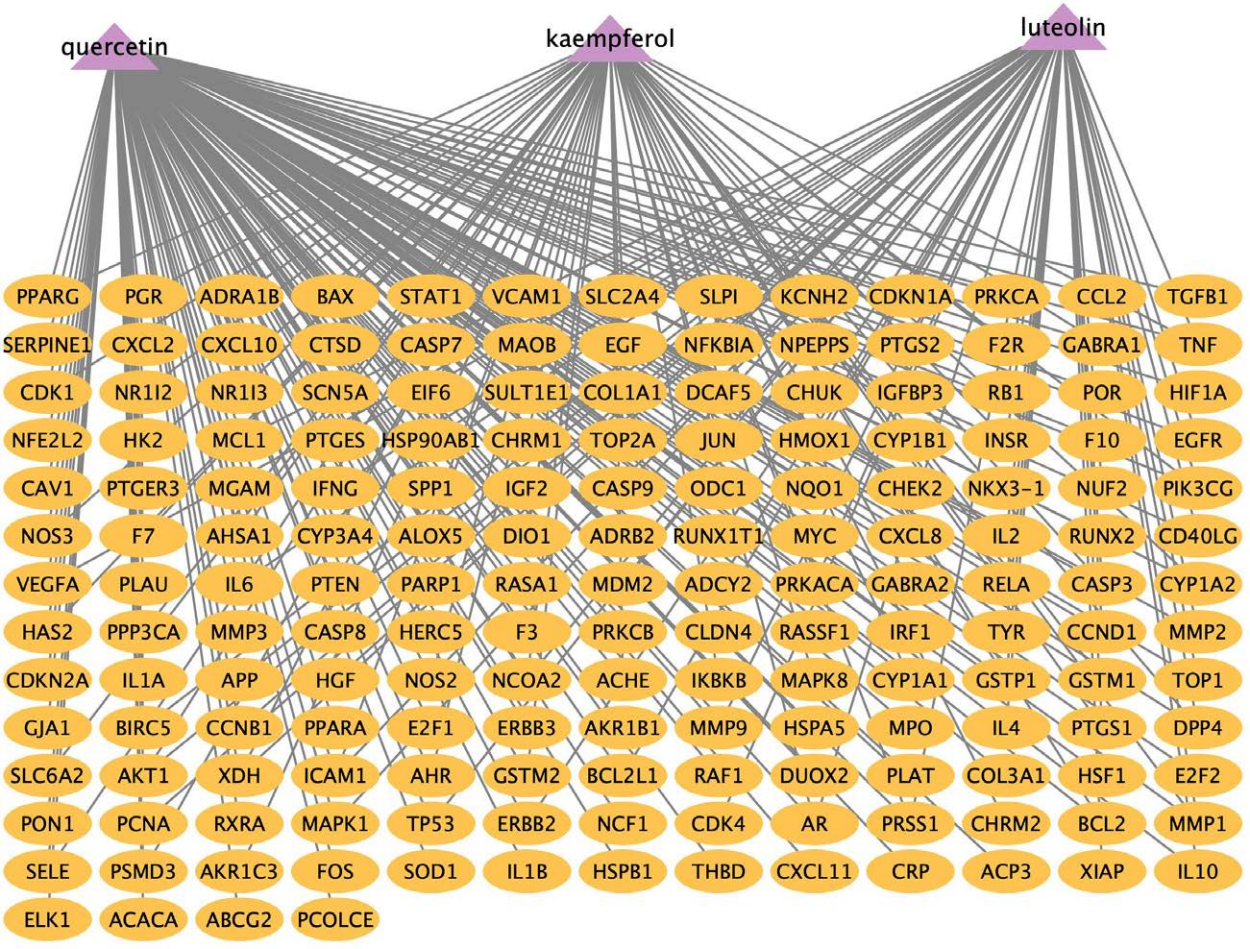
Active compounds – AS targets network diagram. Notes: oval shapes in orange represent the targets, triangle shapes in purple represent the 3 active compounds. AS = atherosclerosis.

AS in SMD as well as the corresponding active compounds were inputted into Cytoscape 3.9.1 software to build “active compounds-AS-targets” network diagram. After analysis of the mapping using the Network Analysis plug-in, it was found that the top 3 core active compounds with the degree value from high to low were MOL000098-quercetin, MOL000422-kaempferol, and MOL000006-luteolin. The topological parameters of the active core compounds mentioned above in the network of “active compounds-AS targets” are shown in Figure 4 and Table 2. In the figure, there are 176 nodes (3 active compounds, 217 targets) and 263 edges, oval shapes in orange represent the targets, triangle shapes in purple represent the 3 active compounds.

**Table 2:**
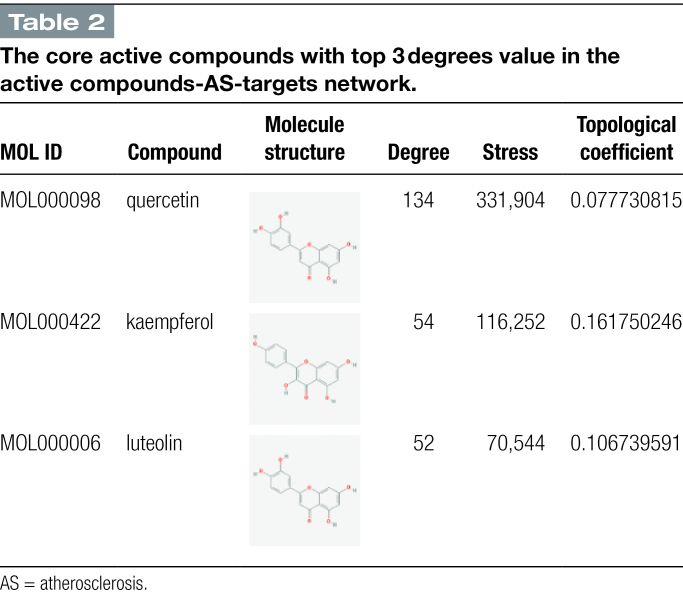
The core active compounds with top 3 degrees value in the active compounds-AS-targets network.

### 3.4. PPI Network.

**Figure 5.**
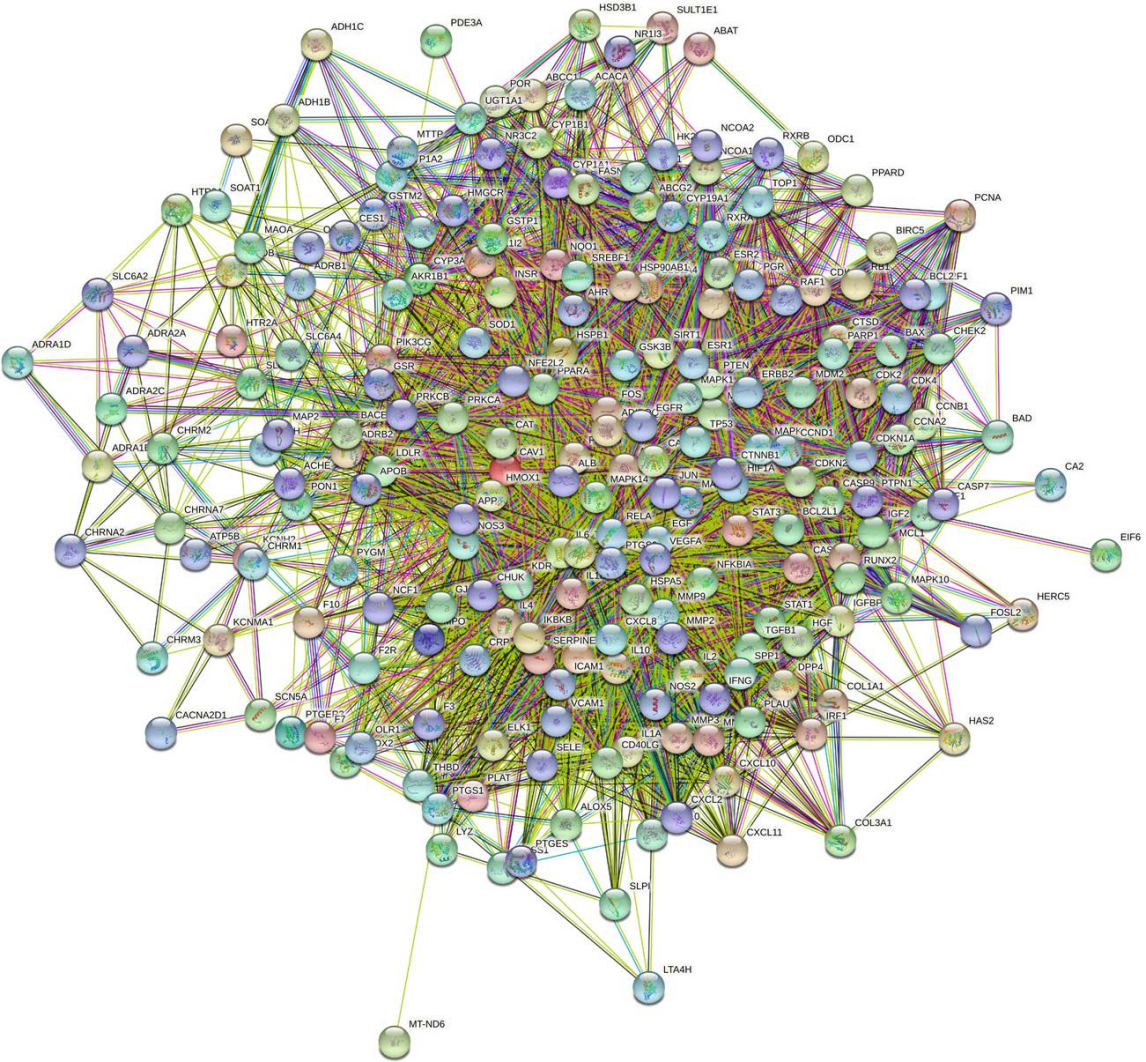
The PPI network of 217 targets of SMD intervening AS. AS = atherosclerosis, PPI = protein–protein interaction, SMD = Simiao Decotion.

**Figure 6.**
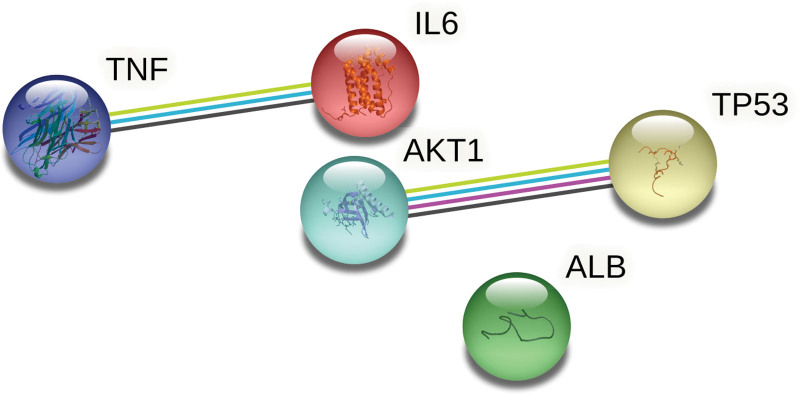
The PPI network of 5 core targets. PPI = protein–protein interaction.

As seen in Figure 5, the PPI network was recreated after being taken from the STRING.11.5 database. The PPI network diagram has 216 nodes (target proteins) and 4807 edges (protein interactions). One target of SMD-AS has not been identified. The scale of degree value is represented by the node’s color. As shown in Table 3, the outcomes of the network topology study revealed that the 5 targets chosen based on degree value were albumin (ALB), AKT1, TNF, IL6, and TP53. The STRING.11.5 database was then set to “highest confidence 0.9” in order to create a PPI network with 5 core targets (Fig. 6).

**Table 3:** Topological parameters of the core targets in PPI.

Gene_Symbol	Degree	Stress	Clustering coefficient	Topological coefficient
ALB	151	39,062	0.33581	0.263136721
AKT1	150	35,586	0.34586	0.264775194
TNF	138	26,572	0.37755	0.277437572
IL6	134	27,094	0.3866	0.282878063
TP53	130	17,434	0.42612	0.301923077

PPI = protein–protein interaction.

### 3.5. Biological function and pathway.

**Figure 7.**
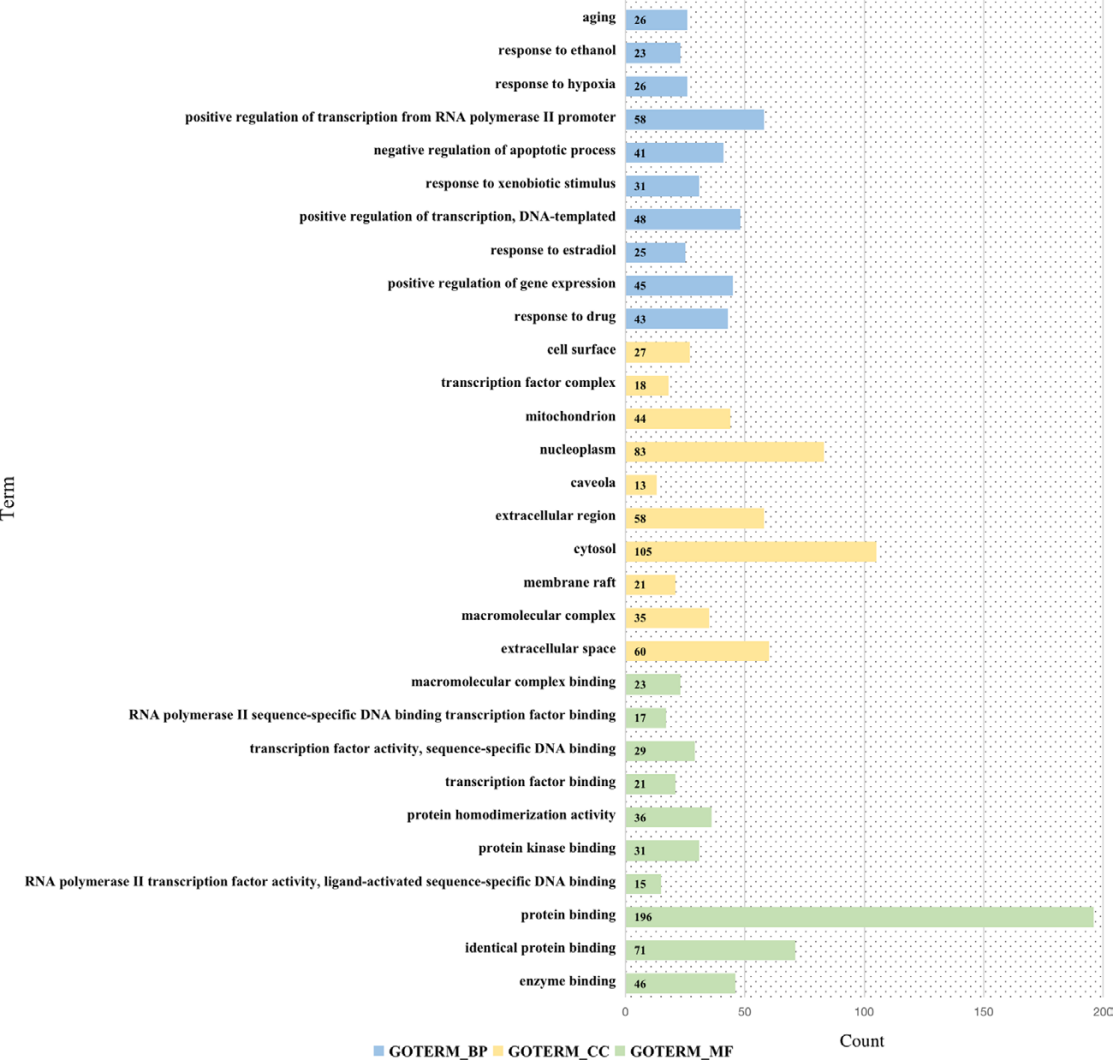
The bar chart of the GO functions of the AS targets mediated by SMD. Notes: The light blue indicates BP, light yellow indicates CC, and light green indicates MF. AS = atherosclerosis, BP = biological processes, CC = cell compositions, GO = Gene Ontology, MF = molecular functions, SMD = Simiao Decotion.

The results of the analysis of the GO function showed that 991 enrichment results were obtained after screening (*P* < .05), including 751 biological processes (BP), 88 cell compositions (CC), and 152 molecular functions (MF).^[21]^ In the study, the top 10 of BP, MF, and CC results were screened as per *P*-value for mapping a histogram, as shown in Figure 7, where BP mainly covered positive regulation of transcription from RNA polymerase II promoter, positive regulation of gene expression, and response to drugs, estradiol, exogenous stimulation, etc. CC generally were related to extracellular space, cytosol, and nucleoplasm. MF mainly included protein binding and enzyme binding.

**Figure 8.**
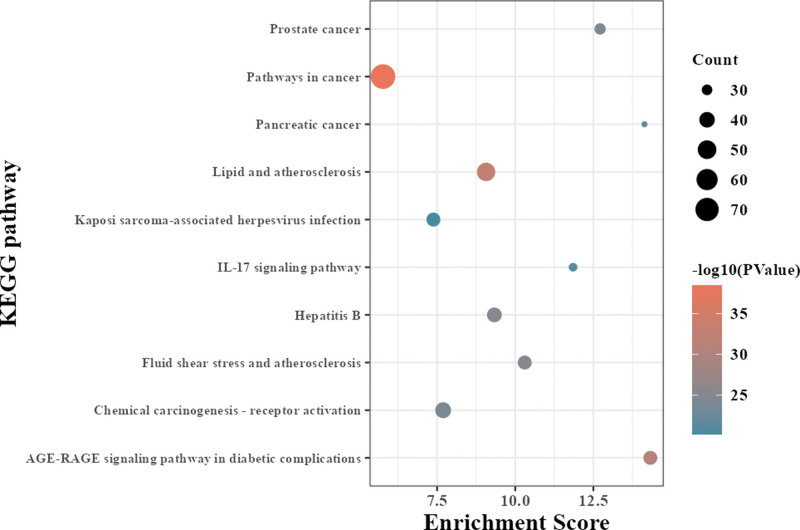
KEGG pathway bubble chart of SMD intervening AS targets. AS = atherosclerosis, KEGG = Kyoto Encyclopedia of Genes and Genomes, SMD = Simiao Decotion.

KEGG analysis showed that 169 signaling pathways (*P* < .05) were involved in Pathways in cancer, Lipid and AS, Chemical carcinogenesis - receptor activation, advanced glycosylation end product-receptor of AGE (AGE-RAGE) signaling pathway in diabetic complications, and Hepatitis B, etc. In the study, the top 10 signal pathways were selected according to the *P* value to map a path bubble diagram, as shown in Figure 8.

### 3.6. Molecular docking.

**Figure 9.**
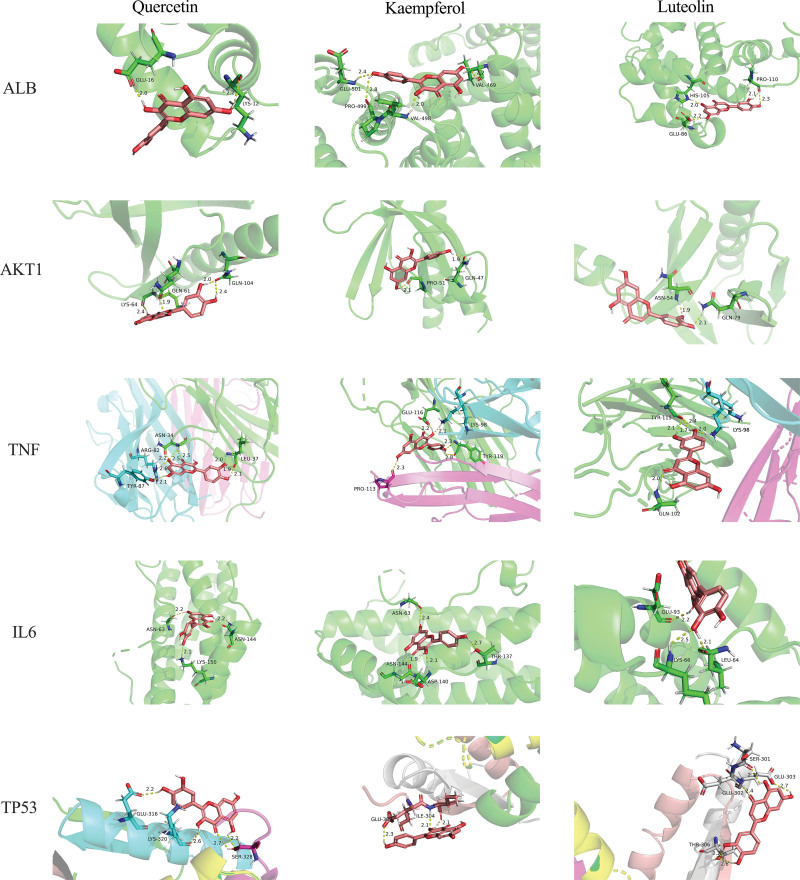
Molecular docking diagram of the top 3 active compounds and the top 5 targets.

**Figure 10.**
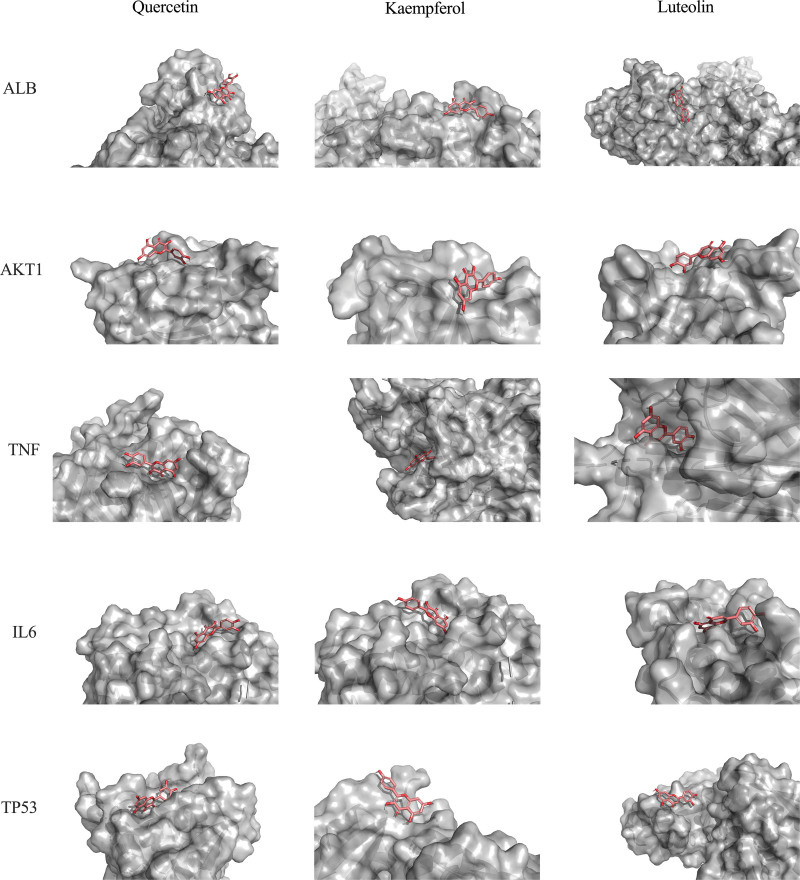
Each ligand receptor binding region.

The protein receptors and active compounds corresponding to ALB (PID:1N5U), AKT1 (PID: 1H10), TNF (PID:7KP9), IL6 (PID: 1ALU), and TP53 (PID: 4CZ7) were selected from the RSCB PDB database and PubChem database for molecular docking. The docking results of the 3 active core compounds and the 5 core protein receptors were all less than −10.0 kJ/mol, suggesting that the active core compounds in the SMD and core targets in AS had good binding capacity, respectively shown in Table 4 and Figures 9 and 10. The lower the binding energy of small molecule ligands and protein receptors, the more stable the binding molecules are, indicating that the possibility of interaction between the molecules is greater.

**Table 4:** The binding energy of core active compounds and coreprotein receptors.

Compound	Binding energy (kJ/mol)				
	ALB	AKT1	TNF	IL6	TP53
quercetin	−10.62736	−13.34696	−15.56448	−11.0876	−13.89088
kaempferol	−14.14192	−14.14192	−12.84488	−13.55616	−14.14192
luteolin	−12.51016	−14.4348	−15.31344	−13.05408	−15.43896

## 4. Discussion

AS is a condition characterized by thickening of the artery wall or functional degeneration, including endothelial cell destruction, issues with lipid metabolism, and infiltration of inflammatory cells.^[24,25]^ According to several research, Simiao Yong’an Decoction’s active components have an anti-AS effect,^[26]^ and the ingredients in the prescription are similar to those in SMD. Therefore, finding the target proteins and potent active compounds in SMD to treat AS is extremely important.

This study’s major goal is to investigate SMD’s possible therapeutic mechanism for AS. 124 active compounds were gathered from the TCMSP database, including luteolin, kaempferol, and quercetin, which can interact with other active substances to operate on a variety of network targets. Quercetin has been shown to prevent the growth of low-density lipoprotein (ox-LDL)-induced foam cells and slow down the aging process by causing RAW264.7 cells to engage in STE20 like kinase (MST1)-mediated autophagy.^[27]^ Quercetin inhibits the Apoe^−/−^ mouse AS model’s ability to control the expression of proprotein convertase subtilisin/kexin type 9, CD36, PPAR, liver X receptor, and ATP-binding cassette transporter A1 in order to avoid AS.^[28,29]^ Kaempferol can cure AS by preventing the death of endothelial cells.^[30]^ Moreover, by controlling the genes and proteins of the related inflammatory factors E-selectin, intercellular adhesion molecule-1, vascular cell adhesion molecule-1, and monocyte chemoattractant protein-1, kaempferol can also prevent AS in the rabbit model.^[31]^ Luteolin’s anti-inflammatory properties were demonstrated by its ability to prevent the activation of the nucleotide oligomerization domain-like receptor thermal protein domain associated protein 3 inflammatory body and to encourage the polarization of macrophages to the M2 phenotype.^[32]^ By controlling the inflammatory response, which is mediated by signal transducer and activator of transcription 3, luteolin can help reduce AS.^[33]^ The aforementioned results imply that these active components may be crucial for SMD’s therapeutic impact on AS and demand further study.

We gathered disease targets for AS in the DrugBank, OMIM, and TTD databases. We detected 217 shared targets between AS and SMD. These targets are thought to be possible AS and SMD therapy targets. We built a PPI network expressing protein-protein interaction to investigate the key target. According to the PPI network data, the main targets may include ALB, AKT1, TNF, IL6, and TP53. ALB performs physiological tasks include transporting and binding both endogenous and foreign materials, preserving the osmotic pressure of plasma colloid, neutralizing free radicals, and influencing the permeability of arterial blood vessels. Advanced glycosylated ALB can cause endoplasmic reticulum dysfunction, which can increase the risk of AS.^[34]^ AKT may control numerous downstream processes simultaneously, and after PI3K activation, AKT kinase activity is increased in a variety of growth factor receptor-mediated signal cascades.^[35]^ Cell growth and proliferation are mostly regulated by the PI3K-AKT signal pathway, which frequently collaborates with other signal pathways in this regard.^[36]^ It has been demonstrated that TNF-related receptor inhibitors enhance vascular endothelial function and decrease the concentration of serum adhesion molecules^[37]^ IL-6 is an inflammatory cytokine that can induce the death and necrosis of smooth muscle cells and vascular endothelial cells, which can result in the development and rupture of atherosclerotic plaque and even thrombosis.^[38]^ By inhibiting TP53 expression, miR-652-3p regulates the lipid metabolism of macrophages to reduce AS.^[39]^

We used GO analysis and KEGG enrichment analysis to investigate the mechanism of SMD in the treatment of AS. The GO findings revealed that the target genes were mostly rich in biological activities, BP mainly covered positive regulation of transcription from RNA polymerase II promoter, positive regulation of gene expression, and response to drugs, estradiol, exogenous stimulation, etc. CC generally were related to extracellular space, cytosol, and nucleoplasm. MF mainly included protein binding and enzyme binding. Not only is catalytically active gamma-glutamyl transferase present in atherosclerotic plaques, but there is also a correlation between gamma-glutamyl transferase activity and markers of plaque instability, suggesting that the enzyme plays a direct role in the pathophysiology of AS and associated clinical events by encouraging pro-oxidant reactions.^[40]^ KEGG enrichment analysis results show that Pathways in cancer, Lipid and AS, Chemical carcinogenesis - receptor activation, AGE-RAGE signaling pathway in diabetic complications, and Hepatitis B, etc. AS and tumorigenesis may be linked by lectin-like oxidized ox-LDL receptor-1, indicating that inhibiting lectin-like oxidized ox-LDL receptor-1 may be a promising approach to treating both AS and malignancies.^[41]^ Via the suppression of oxidative stress, a combination of vitamin D receptor and retinoid X receptor agonists successfully treated diabetic AS, and it is possible that vitamin D receptor activation is partially responsible for the preventative actions of retinoid X receptor agonist.^[42]^ Peroxisome proliferator-activated receptor α, a nuclear receptor, can lessen AS and inhibit the development of macrophage foam cells, vascular inflammation, vascular smooth muscle cell migration and proliferation, plaque instability, and thrombosis.^[43]^ By activating Nox-1, nuclear factor κB, and other signaling pathways, the AGE-RAGE signaling pathway in diabetes complications can increase the production of reactive oxygen species, promote oxidative stress, and induce the occurrence and development of AS. Conversely, reducing the production of reactive oxygen species can decrease vascular calcification.^[44]^ Untreated HBeAg-negative chronic Hepatitis B is a unique risk factor for subclinical AS and carotid plaques.^[45]^

Based on the above results of network pharmacological research, specifically the screening results of compound active ingredients and the screening results of targets in PPI network, we screened 3 important bioactive components and 5 representative targets in order to investigate the potential molecular mechanism of SMD in treating AS. ALB, AKT1, TNF, IL6, and TP5 protein molecules are docked with quercetin, kaempferol, and luteolin, respectively, to confirm our network pharmacological prediction results. The docking findings revealed that all 3 substances could attach to protein with good efficiency, with quercetin and TNF having the lowest binding energy and the most stable combination. It can be used as a further research direction.

There are still some limitations to this study. In this study, we investigate the targets of SMD in treatment of AS using network pharmacology and molecular docking techniques, the only modern bioinformatics methods. Quercetin, kaempferol, and luteolin have been identified as the 3 most significant bioactive components of SMD in treating AS, although they are unable to fully represent SMD. ALB, AKT1, TNF, IL6, and TP53 are also the primary targets.

Some of the results of our research are less in previous studies. However, the accuracy and timeliness of database data need to be scientifically confirmed, and the present network information technology has to be further enhanced. We might not include in our study any chemicals or targets that have not been verified and recorded. In addition, we must continue to think about pharmacodynamic studies. We think there is a lot of space for development and study because the effects and mechanisms of these possible active components on AS haven’t been clarified and proven.

## 5. Conclusion

Based on network pharmacology and molecular docking technology, this study conducted a preliminary exploration of the main chemical components and potential mechanism of SMD in the treatment of AS. Our research shows that quercetin, kaempferol, and luteolin may be the main active ingredients of SMD, and the protein target ALB, AKT1, TNF, IL6, and TP53 may be a potential therapeutic target of SMD in the treatment of AS. And it may play a therapeutic role through Pathways in cancer, Lipid and AS, Chemical carcinogenesis - receptor activation, AGE-RAGE signaling pathway in diabetic complications, and Hepatitis B, etc. In addition, our study will provide a reference for further research on the mechanism of SMD in the treatment of AS.

## Acknowledgements

We thank Yihui Chai for article revision/review; Wen Li and Liancheng Guan for assistance in data curation; and Yizi Fan for help with visualization.

## Author contributions

**Data curation:** Qian Li, Wen Li, Liancheng Guan.

Funding acquisition: Yunzhi Chen.

Project administration: Yunzhi Chen.

Visualization: Yizi Fan.

Writing – original draft: Qian Li.

Writing – review & editing: Yihui Chai.
